# Elevated Plasma C-Terminal Endothelin-1 Precursor Fragment Concentrations Are Associated with Less Anxiety in Patients with Cardiovascular Risk Factors. Results from the Observational DIAST-CHF Study

**DOI:** 10.1371/journal.pone.0136739

**Published:** 2015-08-31

**Authors:** Thomas Meyer, Mira-Lynn Chavanon, Christoph Herrrmann-Lingen, Maren Roggenthien, Kathleen Nolte, Burkert Pieske, Rolf Wachter, Frank Edelmann

**Affiliations:** 1 Department of Psychosomatic Medicine and Psychotherapy, University of Göttingen, Göttingen, Germany; 2 Department of Cardiology and Pneumology, University of Göttingen, Göttingen, Germany; 3 Department of Internal Medicine and Cardiology, Charité University Medicine, Berlin, Germany; 4 German Centre for Cardiovascular Research, partner site Göttingen, Göttingen, Germany; 5 German Center for Cardiovascular Research, partner site Berlin, Berlin, Germany; Scuola Superiore Sant'Anna, ITALY

## Abstract

**Background:**

The role of endothelin-1 (ET-1) in the neurobiology of anxiety is unknown, therefore, we assessed in the observational multicenter DIAST-CHF study whether the C-terminal ET-1 precursor fragment (CT-proET-1) is linked to anxiety.

**Methods:**

Plasma concentrations of CT-proET-1 were measured in a total of 1,410 patients presenting with cardiovascular risk factors (mean age 66.91±8.2 years, 49.3% males, mean left ventricular ejection fraction 60.0±8.2%) who had completed the Hospital Anxiety and Depression Scale (HADS) questionnaire.

**Results:**

Among the total study cohort (n = 1,410), there were 118 subjects (8.4%) with an HADS anxiety score above the cut-off level of 11 suggestive of clinically relevant anxiety. Plasma CT-proET-1 levels were significantly lower in the group of anxious patients as compared to non-anxious patients (p = 0.013). In regression models adjusted for sex, age, systolic blood pressure, and diameters of left atrium and ventricle, plasma CT-proET-1 was again linked to anxiety (Exp(β) = 0.247, 95%-confidence interval [95%-CI] = 0.067–0.914, p = 0.036). Given the high prevalence of depressive disorders in anxious patients, we additionally included the HADS depression score as an independent variable in the models and found that CT-proET-1 remained a significant predictor of anxiety, independent of comorbid depression (Exp(β) = 0.114, 95%-CI = 0.023–0.566, p = 0.008).

**Conclusions:**

Our data from a population-based study in outpatients with cardiovascular risk factors revealed that circulating CT-proET-1 levels are negatively associated with anxiety. Further investigations are required to clarify the putative anxiolytic effect of ET-1 or its precursor molecules in humans and to decipher its mechanistic pathways.

## Introduction

Epidemiological evidence supports a pathological link between emotional distress and cardiovascular morbidity and mortality. Research on cardiovascular patients has largely focused on depression, whereas the impact of anxiety on pathways linking subjective perception of stress to the regulation of cardiovascular homeostasis has been less well studied. In this respect, neuroendocrine markers expressed both in the brain and the vasculature are of particular interest, since they may affect emotional functioning or trigger mood disorders, in addition to their better-defined peripheral vasoactive effects. It has already been shown that elevated plasma levels of pro-atrial natriuretic peptide are associated with low anxiety, suggesting a negative feedback loop limiting psychological distress and its adverse autonomic consequences in patients with heart failure [1]. Whether the 21-amino acid neuropeptide ET-1 is also linked to anxiety states has not been investigated so far. However, two studies have reported a positive association between circulating ET-1 levels and depressive symptom severity in patients with coronary artery disease [2,3], indicating that this vasoactive peptide modulates brain functions [4]. In addition, it was shown that ET-1 decreases the high-affinity uptake of the excitatory amino acid glutamate in primary cultured astrocytes from rat cerebral cortex, suggesting a direct effect on membrane depolarisation [5]. This is of special interest as a growing body of evidence suggests that glutamatergic neurotransmission may be involved in the biological mechanisms underlying stress response, panic/anxiety and anxiety-related disorders [6,7]. Recently, initial evidence has indicated that even sub-clinical anxiety is associated with altered glutamate metabolism [8]. Given these links between glutamatergic neurotransmission, stress response and ET-1, we wondered whether there is any evidence of an association between anxiety and the ET-1 signal pathway. In a sample of patients with cardiovascular risk factors, we therefore tested the possible relationship between plasma concentrations of the stable endothelin precursor fragment CT-proET-1 and anxiety.

## Methods

### Subjects

Medical outpatients aged 50 to 85 years presenting with cardiovascular risk factors were recruited for the Diagnostic Trial on Prevalence and Clinical Course of Diastolic Dysfunction and Heart Failure (DIAST-CHF) study. The multicenter, population-based DIAST-CHF study was part of the nationwide German Competence Network Heart Failure, which was initiated in 2004 and financed by the German Ministry of Education and Research [9–11]. A network of primary care physicians referred study candidates for further cardiological and psychometric assessment when at least one risk factor for the development of diastolic dysfunction was detected at the screening visit. Inclusion criteria for participation in the study were physician-diagnosed and/or medically treated hypertension, diabetes mellitus, coronary heart disease, sleep apnea syndrome and/or history of heart failure [9–11]. Patients were excluded from the study when unwilling to give informed consent and in the case of insufficient understanding of the German language and/or unavailability for logistic reasons. The study protocol was approved by the following local institutional ethics committees from the academic centers which participated in the study: medical faculties of the Universities of Berlin, Essen, Göttingen, Lübeck, Marburg, and Würzburg. The study complies with the Declaration of Helsinki, and all patients gave written informed consent before being included in the study.

### Clinical procedures

After study enrolment, all patients received a routine physical examination and a detailed cardiological assessment. Blood pressure was measured using an automated auscultatory technique, and transthoracic echocardiography was performed by experienced physicians on a Hewlett-Packard Sonos 5500 (Hewlett-Packard, Andover, MA, USA). Standardized imaging planes in parasternal (long- and short-axis) and apical (2- and 4-chambers) views were used for quantifying cardiac chamber dimensions. All examinations included B- and M-mode with colour Doppler. The clinical severity of heart failure was rated according to the Framingham sum score [12]. The cardiologists, who performed the physical and echocardiographical examinations, were blind to the CT-proET-1 levels and the results of the psychometric testing.

### Screening for anxiety and depression

Study participants were requested to complete the German version of the Hospital Anxiety and Depression Scale (HADS) as part of the psychological assessment. This self-assessment questionnaire originally developed for physically ill patients is widely used for screening purposes [13–15]. The instrument comprises 14 four-point Likert-scaled items, seven of which relate to anxiety symptoms (HADS-A) and seven to cognitive-affective features of depression (HADS-D). Each item is coded from 0 to 3 and hence subscale scores range from 0 to 21 with higher scores indicating more severe symptoms. In accordance with the literature, a cut-off level of ≥11 was used to distinguish anxious from non-anxious subjects [13]. The German version of the HADS has been validated extensively and shows good reliability and internal consistency with Cronbach’s α of approximately 0.80 for each of the two subscales.

### Biochemical parameters

From each subject, peripheral venous blood samples were drawn after 15 min of rest into tubes containing ethylenediaminetetra-acetic acid (EDTA). Blood samples were immediately stored on ice and centrifuged within 4 hours. After centrifugation, plasma aliquots were frozen at -80°C until further use. The plasma concentration of CT-proET-1 (amino acids 168–212 of pre-proET-1) was measured using an automated immunoluminometric assay on a Kryptor system (B.R.A.H.M.S AG, Henningsdorf, Germany). This immunoassay which is based on a sandwich technique has been shown to accurately quantify circulating CT-proET-1 levels without the need of preanalytical procedures such as prior extraction [16]. The analytical performance of CT-proET-1 was shown to be superior to ET-1, since a reliable measurement of ET-1 in blood samples is difficult due to its short plasma half-life (1–2 min) which results from the fast clearance attributable to receptor binding during pulmonary passage and cleavage by neutral endopeptidases [16]. Papassotiriou et al. reported that the CT-proET-1 assay allows precise measurement of the analyte with good assay linearity as assessed by serial dilution experiments [16]. The intra-assay and inter-assay coefficients of variability (CV) were less than 10% for all investigated samples, including health controls, and the analytical detection limit was 0.4 pmol/L [16]. High-sensitivity C-reactive protein was determined using a standard immunoturbidometric assay on Cobas INTEGRA (Roche Diagnostics) [17]. Circulating natriuretic peptides were analyzed using commercially available immunoluminometric kits: the pro-ANP microtiter immunoassay was purchased from Immundiagnostik (Freiburg, Germany), mid-regional pro-atrial natriuretic peptide (MR-proANP) was measured using the BRAHMS Seristra kit (Henningdorf, Germany), brain natriuretic peptide (BNP) was determined using the ADVIA Centaur BNP assay (Bayer Diagnostics, Munich, Germany), and amino-terminal pro-brain natriuretic peptide (NT-proBNP) was measured on a Cobas 8000 or Cobas e411 analyser (Roche Diagnostics, Mannheim, Germany) [1].

### Statistical analyses

Demographic and clinical data are presented as means and standard deviations or frequencies and percentages. Plasma CT-proET-1 concentrations are given as medians and interquartile ranges (IQR). Student’s *t* or Fisher’s exact test was used to compare the two groups of study participants with and without elevated HADS-A scores, while the Mann-Whitney U-test was used to compare distributions of the non-normally distributed CT-proET-1 concentrations between the two groups. Logistic regression models with an elevated HADS-A score as dependent variable and CT-proET-1 levels as independent variables were computed adjusted for all potential confounders which in bivariate analyses have proven to be significantly associated with clinically relevant anxiety. Regression analyses with CT-proET-1 concentrations were calculated with log10-transformed values. Given the association between depressive mood and comorbid anxiety, we additionally controlled for depressive symptoms by entering HADS-D scores as an independent variable. Data are presented as multivariate odds ratios (exp(β)-coefficients) and their 95%-confidence intervals (95%-CI). In order to test whether the presumed association between CT-proET-1 and anxiety depends on antihypertensive drug treatment, we specified separate simple moderation models using the SPSS macro PROCESS v2.10 by Hayes [18]. Using ordinary least square (OLS) regression, different classes of antihypertensive drugs were probed for interaction by means of the 95% percentile-based confidence interval. Similar moderation models were computed for circulating neuropeptides and CRP using HADS anxiety as outcome measure. In order to keep the results comparable with those of the regression analyses mentioned above, the covariates entered into the moderation models were kept the same as in the main analyses. All moderation analyses used continuous anxiety scores instead of a dichotomous classification as the to-be-predicted outcome. In all analyses, a p value <0.05 was used to indicate statistical significance. Data were analysed on a personal computer with SPSS 22 for Windows (SPSS Inc., Chicago, IL, USA).

## Results

### Characterization of the study population

A total of 1,410 study participants with available plasma CT-proET-1 levels and HADS scores were included in this analysis. The mean age of the study population was 66.1±8.2 years. Due to the inclusion criteria and study design, the percentage of patients with hypertension (80.7%), hyperlipoproteinemia (40.9%), diabetes mellitus (24.4%), hyperuricemia (14.9%) and current smoking status (29.8%) among the study population was comparably high. The mean HADS-A anxiety score was 5.0±3.6, while the median of the CT-proET-1 concentration was 55.5 pmol/l with an IQR from 47.6 to 65.1 pmol/l.

### Differences between the groups of anxious versus non-anxious participants

As expected, anxious patients differed from non-anxious study participants in that they were more likely to be female (64.4% versus 49.5%, p = 0.003) and of younger age (62.6±7.4 versus 66.4±8.2 years, p<0.001, Table 1). The Framingham sum score was significantly higher in anxious patients as compared to non-anxious subjects (1.15±1.27 versus 0.71±1.12, p<0.001), while their mean systolic blood pressure was lower (142.2±19.5 mmHg versus 147.6±21.2 mmHg, p = 0.008). The mean left-ventricular end-diastolic diameter (LVEDD, 48.3±5.0 mm versus 49.5±6.3mm, p = 0.018) and left-atrial diameter (LA, 40.1±6.0 mm versus 41.5±6.3 mm, p = 0.026) were both significantly lower in anxious subjects than in the non-anxious group. However, the two groups did not significantly differ with respect to the prevalence of cardiovascular risk factors, such as hypertension, diabetes mellitus, hyperuricemia, smoking status and sleep apnea (Table 1). Likewise, there was no significant difference in body-mass index, heart rate, history of myocardial infarction, atrial fibrillation and 6-min walking distance between the groups of anxious versus non-anxious participants.

**Table 1:** Comparison of the two groups of anxious versus non-anxious patients. Results are presented as means and standard deviations or frequencies, except for serum CT-proET-1 concentrations which are given as medians and interquartile ranges.

	Non-anxious study participants (n = 1292)	Anxious study participants (n = 118)	P value
Sex (male, %)	50.5	35.6	0.003
Age (years)	66.4±8.2	62.6±7.4	<0.001
Hypertension (%)	80.5	83.1	0.545
Hyperlipoproteinemia (%)	40.7	42.4	0.769
Diabetes mellitus (%)	23.9	29.7	0.179
Hyperuricemia (%)	15.4	9.3	0.080
Current smoking (%)	10.1	15.3	0.085
Sleeping apnea (%)	6.2	5.9	1.000
Body-mass index (kg/m^2^)	28.9±4.9	29.8±5.3	0.071
6-min walking distance (m)	517±106	513±106	0.702
Framingham sum	0.71±1.12	1.15±1.27	<0.001
Atrial fibrillation (%)	7.1	3.4	0.178
History of MI (%)	9.3	9.3	1.000
Systolic BP (mmHg)	147.6±21.2	142.2±19.5	0.008
Diastolic BP (mmHg)	83.2±11.8	83.4±12.2	0.859
Heart rate (bpm)	70.3±11.7	70.0±11.6	0.774
LA (mm)	41.5±6.3	40.1±6.0	0.026
LVESD (mm)	31.2±6.4	30.8±5.3	0.477
LVEDD (mm)	49.5±6.3	48.3±5.0	0.018
LVEF (%)	59.9±8.3	61.0±7.0	0.186
HADS-A score	4.3±2.9	12.6±1.6	<0.001
HADS-D score	3.7±3.2	9.5±3.5	<0.001
HADS-D ≥8 (%)	12.1	69.5	<0.001
CT-proET-1 (pmol/l)	55.6 (47.9–65.2)	52.6 (44.2–62.7)	0.013

Abbreviations: HADS-A; anxiety subscale of the Hospital Anxiety and Depression Scale, LA; left atrium; LVEDD; left ventricular end-diastolic diameter, LVESD; left ventricular end-systolic diameter, LVEF; left ventricular ejection fraction.

### Association of plasma CT-proET-1 levels with anxiety

In univariate analysis, we found that the group of non-anxious participants had a significantly higher median CT-proET-1 level (55.6 pmol/l, IQR = 47.9–65.2) as compared to anxious subjects (52.6 pmol/l, IQR = 44.2–62.7, r = 0.066, p = 0.013). Based on this observation, we computed a set of multivariate logistic regression models with clinically elevated HADS-A scores as the dependent variable adjusted for all potential confounders which in univariate analyses had proven to be significantly different between the two groups of anxious and non-anxious subjects. When adjusted for sex, age, BMI, systolic blood pressure, LA and LVED diameter, and Framingham sum score, we found that plasma CT-proET-1 was again significantly and independently associated with lower anxiety (exp(β) = 0.25, 95%-CI = 0.07–0.91, p = 0.036, Table 2).

**Table 2:** Results from logistic regression models with clinically relevant anxiety as dependent variable adjusted for the confounders indicated. In models 2 and 3, CT-proET-1 was included as an independent variable and in model 3, HADS-D was additionally entered as a covariate.

A				
Model 1: Dependent variable HADS-A≥11 (total model p<0.001, R^2^ = 0.042)	Exp(β)	95%-CI	Wald value	P value
Sex	1.408	0.898–2.208	2.227	0.136
Age	0.934	0.909–0.959	25.354	<0.001
Systolic blood pressure	0.994	0.984–1.003	1.683	0.195
Left atrial diameter (mm)	0.985	0.949–1.023	0.605	0.437
LVEDD (mm)	0.975	0.939–1.013	1.711	0.191
Framingham sum	1.448	0.898–1.675	24.709	0.136
B				
Model 2: Dependent variable HADS-A≥11 (total model p<0.001, R^2^ = 0.045)	Exp(β)	95%-CI	Wald value	P value
Sex	1.417	0.902–2.224	2.288	0.130
Age	0.939	0.914–0.965	20.106	<0.001
Systolic blood pressure	0.993	0.983–1.002	2.191	0.139
Left atrial diameter (mm)	0.990	0.954–1.028	0.286	0.593
LVEDD (mm)	0.974	0.938–1.012	1.794	0.180
Framingham sum	1.486	1.279–1.725	26.921	<0.001
Log (CT-proET-1 concentration)	0.247	0.067–0.914	4.386	0.036
C				
Model 3: Dependent variable HADS-A≥11 (total model p<0.001, R^2^ = 0.182)	Exp(β)	95%-CI	Wald value	P value
Sex	2.017	1.176–3.460	6.497	0.011
Age	0.956	0.927–0.986	8.221	0.004
Systolic blood pressure	0.996	0.985–1.007	0.496	0.481
Left atrial diameter (mm)	0.971	0.929–1.014	1.800	0.180
LVEDD (mm)	0.995	0.954–1.038	0.055	0.815
Framingham sum	1.188	0.990–1.424	3.446	0.063
HADS depression	1.496	1.403–1.595	151.539	0.000
Log (CT-proET-1 concentration)	0.114	0.023–0.566	7.061	0.008

Abbreviations: 95%-CI; 95%-confidence interval, HADS; Hospital Anxiety and Depression Scale, LVEDD; left ventricular end-diastolic diameter.

Given the established relationship between depression and comorbid anxiety, we finally tested whether plasma CT-proET-1 concentration may still predict anxiety when additionally controlling for HADS depression. As demonstrated in Table 2 (model 3), the inclusion of HADS-D in this model did not change the significant association between CT-proET-1 and anxiety (exp(β) = 0.11, 95%-CI = 0.02–0.57, p = 0.008). While the corrected R^2^ of this final model increased as expected, CT-proET-1 remained a significant predictor of clinically relevant anxiety, independent of comorbid depression as measured by the HADS-D depression score. In addition, we confirmed that CT-proET-1 is positively associated with depression severity when adjusted for sex and age (β = 2.0, 95%-CI = 0.5–3.4, p = 0.007), however, this significance is lost as long as the Framingham sum score is included in these models (data not shown).

### Control analyses: Antihypertensive treatment and circulating natriuretic peptides as possible moderators

In order to rule out the possibility that the observed magnitude of the relationship between anxiety and CT-proET-1 changes depending upon medication or circulating natriuretic peptides as potential moderators, we probed the respective moderation effects. Analyses showed that neither global (antihypertensive medications: b = -0.71, 95%-CI = -3.20–1.78, p = 0.575 and number of blood pressure-lowering drugs, b = -0.24, 95%-CI = -0.9–0.45, p = 0.487) nor more specific antihypertensive medications (beta blockers, p = 0.838, diuretics, p = 0.293, or angiotensin-converting enzyme/angiotensin-receptor blockers, p = 0.575) moderated the association between CT-proET1 and anxiety.

Because CT-proET-1 was significantly correlated with BNP (r = 0.29), NT-proBNP (r = 0.37), NT-proANP (r = 0.34), MR-proANP (r = 0.45), and CRP (r = 0.20, in all bivariate analyses p<0.001), we next computed a set of regression models by entering each of these parameters as modulators. While CT-proET-1 remained a significant and independent predictor of anxiety in all these models, none of the neurohormones or the inflammation marker CRP, including their interaction terms with CT-proET-1, reached significance (Table 3). Thus, our data support the conclusion that plasma CT-proET-1 levels predict anxiety independent of natriuretic peptides or CRP.

**Table 3:** Moderator analyses of the relationship between anxiety and CT-proET-1 Each model was adjusted for sex, age, systolic blood pressure, left atrial diameter, LVEDD, Framingham sum, and HADS depression using anxiety as outcome measure.

A			
Model 1 (total model p<0.001, R^2^ = 0.466)	Coeff.	95%-CI	P value
C-reactive protein	-0.123	-0.435–0.193	0.445
CT-proET-1	-1.985	-3.019-(-0.951)	<0.001
Interaction term	-0.114	-2.365–2.137	0.921
B			
Model 2 (total model p<0.001, R^2^ = 0.468)	Coeff.	95%-CI	P value
MR-proANP	-0.383	-1.166–0.400	0.337
CT-proET-1	-1.792	-2.896-(-0687)	0.001
Interaction term	-1.370	-5.170–2.430	0.479
C			
Model 3 (total model p<0.001, R^2^ = 0.462)	Coeff.	95%-CI	P value
NT-proANP	-0.382	-1.808–1.044	0.599
CT-proET-1	-2.073	-3.448-(-0.698)	0.003
Interaction term	-4.409	-10.063–1.245	0.126
D			
Model 4 (total model p<0.001, R^2^ = 0.459)	Coeff.	95%-CI	P value
BNP	-0.078	-0.437–0.281	0.670
CT-proET-1	-2.253	-3.456-(-1.051)	<0.001
Interaction term	-1.463	-3.582–0.655	0.176
E			
Model 5 (total model p<0.001, R^2^ = 0.468)	Coeff.	95%-CI	P value
NT-proBNP	-0.293	-0.664–0.078	0.121
CT-proET-1	-1.809	-2.866-(-0.753)	0.001
Interaction term	-0.197	-2.228–1.834	0.849

Abbreviations: ANP; atrial natriuretic peptide, BNP; brain natriuretic peptide, 95%-CI; 95%-confidence interval, Coeff, coefficient; CRP; high-sensitivity C-reactive protein, MR-proANP; mid-regional pro-atrial natriuretic peptide, and NT-proBNP; amino-terminal pro-brain natriuretic peptide.

## Discussion

Previous studies have demonstrated that, in patients with coronary artery disease, the severity of depressive symptoms predicts elevated ET-1 [2,3], therefore, the purpose of the present re-analysis of DIAST-CHF data was to elucidate whether the circulating precursor CT-proET-1 is linked to anxiety, independent of depression. In particular, we aimed to clarify a putative anxiolytic effect due to ET-1, or its precursor molecules or proteolytic fragments, in humans and to decipher its mechanistic pathways. Our main finding is that, in contrast to their reported positive association with depression, plasma CT-proET-1 concentrations are negatively linked to anxiety in a sample of medical outpatients with cardiovascular risk factors for diastolic dysfunction or overt heart failure and that this association is independent of coexisting depressive symptoms.

Endothelin-1, the most abundant isoform of a family of three isopeptides, is expressed in a variety of tissues including the brain [19]. The *endothelin-1* gene encodes the 203 amino-acid precursor prepro-endothelin which is further cleaved by the enzyme furin convertase to a 38 amino-acid peptide, called big-ET-1 [20]. Mature ET-1 is then generated by the action of endothelin-converting enzymes to produce the active 21-amino acid peptide. The physiological effects of ET-1 are transduced by two pharmacologically distinguishable receptor subtypes, the ET_A_ and ET_B_ receptor. While the ET_A_ receptor located mainly on vascular smooth muscle cells mediates potent vasoconstriction, stimulation of the endothelial ET_B_ receptor results in the release of nitric oxide (NO) and prostacyclin which both cause vasodilatation [21]. Apart from its direct vasomotor effects, sub-nanomolar concentrations of ET-1 have been demonstrated to activate macrophages via NF-kappaB and induce the synthesis of pro-inflammatory cytokines in vascular smooth muscle cells, such as tumor necrosis factor-α (TNFα), interleukin-1 (IL-1) and IL-6 [22,23]. Furthermore, stimulation of these cells with TNFα and interferon-γ markedly elevated the expression of mRNA for prepro-ET-1 and the release of ET-1 [24].

Recently, one study demonstrated that bosentan, a mixed endothelin receptor antagonist, induced an antidepressant-like activity in mice with increase in circulating IL-6 levels, while anxiogenic effects were not detected, as judged from the results of an elevated plus maze test [4]. Loria and co-workers showed that maternal separation as an early life stressor reduced the expression of ET_A_ and ET_B_ receptors in mice and exaggerated acute stress-mediated pressor response in adulthood [25]. Endepols and colleagues stereotactically injected the vasoconstrictor ET-1 close to the anterior cerebral artery in rats which resulted in bilateral ischemic infarction [26]. One month after ET-1 injection, the treated rats showed increased exploratory activity and restlessness in addition to decreased food carrying behavior and walking speed in a foraging situation, which may be explained by decreased anxiety. In contrast, Zhang et al. reported that transgenic mice over-expressing ET-1 in endothelial cells showed increased anxiety after transient occlusion of the middle cerebral artery with 7 days reperfusion [27]. However, conclusions drawn from animal studies inducing severe brain damage due to local ET-1 injection are not applicable to healthy humans.

Since both increased CRP concentrations and elevated ET-1 levels have been reported to predict major adverse cardiovascular events and poorer prognosis in patients with underlying arteriosclerotic diseases [28,29], we searched for a possible moderation of the inflammation marker CRP on the interaction between plasma CT-proET-1 and anxiety but failed to show such an indirect effect. Similarly adjusted moderation models for natriuretic peptides also did not contribute to our understanding of the complex pathophysiology of the newly discovered link between ET-1 and anxiety. Furthermore, we found no indication that antihypertensive medication plays a role in this pathway. Thus, we cannot exclude that ET-1, or some of its precursors or biologically active, proteolytic fragments, confers direct anxiolytic effects on the brain without affecting inflammatory and/or vasoactive responses.

Several limitations concerning our main finding of a negative association between CT-proET-1 and anxiety need to be addressed. Our data are based on a cross-sectional analysis which lacks repeated laboratory measurements of ET1 precursor levels over time and, thus, preclude analysis of time-dependent covariates in a longitudinal study design or the establishment of a cause-effect relationship. The small effect size critically limits the clinical and diagnostic implications of our finding, and the results from our study should be interpreted with caution, unless cohort effects are empirically ruled out. In particular, the fact that the two groups of anxious and non-anxious study participants are unbalanced must be acknowledged. Nevertheless, our investigation also has some strength which includes mainly a large and heterogeneous study population with well-defined, complete clinical and echocardiographic data. In addition, we used a commercially available immunoassay with a high diagnostic accuracy and a well-validated psychometric screening instrument. However, the results from this study justify replication in independent patient samples and longitudinal follow-up studies.

In conclusion, our study provides preliminary evidence that clinically relevant anxiety is linked to low plasma levels of CT-proET-1 and that this association is independent of comorbid depression. Moreover, we found that in our study cohort anxiety and depression have opposite associations with circulating endothelin levels, suggesting a rather complex impact of this neuropeptide on emotional and behavioral brain functions. Further research is necessary to confirm and clarify these relationships.

## References

[1] Herrmann-Lingen C, Binder L, Klinge M, Sander J, Schenker W, Beyermann B, et al High plasma levels of N-terminal pro-atrial natriuretic peptide associated with low anxiety in severe heart failure. Psychosom Med 2003; 65:517–22. 12883099 10.1097/01.psy.0000073870.93003.c4

[2] Burg MM, Martens EJ, Collins D, Soufer R. Depression predicts elevated endothelin-1 in patients with coronary artery disease. Psychosom Med 2011;73:2–6. 10.1097/PSY.0b013e3181fdfb25 20947777 PMC3016462

[3] Yammine L, Frazier L, Padhye NS, Burg MM, Meininger JC. Severe depressive symptoms are associated with elevated endothelin-1 in younger patients with acute coronary syndrome. J Psychosom Res 2014;77:430–44. 10.1016/j.jpsychores.2014.07.019 25129849 PMC4252375

[4] Pinho-Ribeiro FA, Borghi SM, Staurengo-Ferrari L, Filgueiras GB, Estanislau C, Verri WA Jr. Bosentan, a mixed endothelin receptor antagonist, induces antidepressant-like activity in mice. Neurosci Lett 2014;560:57–61. 10.1016/j.neulet.2013.12.018 24361136

[5] Leonova J, Thorlin T, Aberg ND, Eriksson PS, Rönnbäck L, Hansson E. Endothelin-1 decreases glutamate uptake in primary cultured rat astrocytes. Am J Physiol Cell Physiol 2001;281:C1495–503. 11600412 10.1152/ajpcell.2001.281.5.C1495

[6] Zwanzger P, Zavorotnyy M, Gencheva E, Diemer J, Kugel H, Heindel W, et al Acute shift in glutamate concentrations following experimentally induced panic with cholecystokinin tetrapeptide. A 3T-MRS study in healthy subjects. Neuropsychopharmacol 2013;38:1648–54. 10.1038/npp.2013.61 PMC3717541 23463151

[7] Harvey BH, Shahid M. Metabotropic and ionotropic glutamate receptors as neurobiological targets in anxiety and stress-related disorders: focus on pharmacology and preclinical translational models. Pharmacol Biochem Behav 2012;100:775–800. 10.1016/j.pbb.2011.06.014 21708184

[8] Modi S, Rana P, Kaur P, Rani N, Khushu S. Glutamate level in anterior cingulate predicts anxiety in healthy humans: a magnetic resonance spectroscopy study. Psychiatry Res 2014;224(1):34–41. 10.1016/j.pscychresns.2014.03.001 25156662

[9] Stahrenberg R, Edelmann F, Mende M, Kockskämper A, Düngen HD, Lüers C, et al The novel biomarker growth differentiation factor 15 in heart failure with normal ejection fraction. Eur J Heart Fail 2010;12:1309–16. 10.1093/eurjhf/hfq151 20837635 PMC2990410

[10] Stahrenberg R, Edelmann F, Mende M, Kockskämper A, Düngen HD, Scherer M, et al Association of glucose metabolism with diastolic function along the diabetic continuum. Diabetologia 2010;53:1331–40. 10.1007/s00125-010-1718-8 20386878 PMC2877336

[11] Edelmann F, Stahrenberg R, Polzin F, Kockskämper A, Düngen HD, Duvinage A, et al Impaired physical quality of life in patients with diastolic dysfunction associates more strongly with neurohumoral activation than with echocardiographic parameters: quality of life in diastolic dysfunction. Am Heart J 2011;161:797–804. 10.1016/j.ahj.2011.01.003 21473981

[12] Ho KK, Pinsky JL, Kannel WB, Levy D. The progression from hypertension to congestive heart failure. J Am Coll Cardiol 1993;22(4 Suppl A):6A–13A. 8376698 10.1016/0735-1097(93)90455-a

[13] Zigmond AS, Snaith RP. The hospital anxiety and depression scale. Acta Psychiatr Scand 1983;67:361–70. 6880820 10.1111/j.1600-0447.1983.tb09716.x

[14] Herrmann C. International experiences with the Hospital Anxiety and Depression Scale. A review of validation data and clinical results. J Psychosom Res 1997;42:17–41. 9055211 10.1016/s0022-3999(96)00216-4

[15] Bjelland I, Dahl AA, Haug TT, Neckelmann D. The validity of the Hospital Anxiety and Depression Scale. An updated literature review. J Psychosom Res 2002;52:69–77. 11832252 10.1016/s0022-3999(01)00296-3

[16] Papassotiriou J, Morgenthaler NG, Struck J, Alonso C, Bergmann A. Immunoluminometric assay for measurement of the C-terminal endothelin-1 precursor fragment in human plasma. Clin Chem 2006;52:1144–51. 16627560 10.1373/clinchem.2005.065581

[17] Edelmann F, Tomaschitz A, Wachter R, Gelbrich G, Knoke M, Düngen HD, et al Serum aldosterone and its relationship to left ventricular structure and geometry in patients with preserved left ventricular ejection fraction. Eur Heart J. 2012;33:203–12. 10.1093/eurheartj/ehr292 21856682

[18] Preacher KJ, Hayes AF. SPSS and SAS procedures for estimating indirect effects in simple mediation models. Behav Res Methods Instrum Comput 2004;36:717–31. 15641418 10.3758/bf03206553

[19] Firth JD, Ratcliffe PJ. Organ distribution of the three rat endothelin messenger RNAs and the effects of ischemia on renal gene expression. J Clin Invest 1992;90:1023–31. 1522210 10.1172/JCI115915 PMC329959

[20] Denault JB, Claing A, D'Orléans-Juste P, Sawamura T, Kido T, Masaki T, et al Processing of proendothelin-1 by human furin convertase. FEBS Lett 1995;362:276–80. 7729512 10.1016/0014-5793(95)00249-9

[21] Böhm F, Pernow J. The importance of endothelin-1 for vascular dysfunction in cardiovascular disease. Cardiovasc Res 2007;76:8–18. 17617392 10.1016/j.cardiores.2007.06.004

[22] Browatzki M, Schmidt J, Kübler W, Kranzhöfer R. Endothelin-1 induces interleukin-6 release via activation of the transcription factor NF-kappaB in human vascular smooth muscle cells. Basic Res Cardiol 2000;95:98–105. 10826501 10.1007/s003950050170

[23] Browatzki M, Pfeiffer CA, Schmidt J, Kranzhöfer R. Endothelin-1 induces CD40 but not IL-6 in human monocytes via the proinflammatory transcription factor NF-kappaB. Eur J Med Res 2005;10:197–201. 15946919

[24] Woods M, Mitchell JA, Wood EG, Barker S, Walcot NR, Rees GM, et al Endothelin-1 is induced by cytokines in human vascular smooth muscle cells: evidence for intracellular endothelin-converting enzyme. Mol Pharmacol 1999;55:902–9. 10220569

[25] Loria AS, D'Angelo G, Pollock DM, Pollock JS. Early life stress downregulates endothelin receptor expression and enhances acute stress-mediated blood pressure responses in adult rats. Am J Physiol Regul Integr Comp Physiol 2010;299:R185–91. 10.1152/ajpregu.00333.2009 20410476 PMC2904153

[26] Endepols H, Mertgens H, Backes H, Himmelreich U, Neumaier B, Graf R, et al Longitudinal assessment of infarct progression, brain metabolism and behavior following anterior cerebral artery occlusion in rats. J Neurosci Methods 2014 [Epub ahead of print] 10.1016/j.jneumeth.2014.11.003 25445057

[27] Zhang X, Yeung PK, McAlonan GM, Chung SS, Chung SK. Transgenic mice over-expressing endothelial endothelin-1 show cognitive deficit with blood-brain barrier breakdown after transient ischemia with long-term reperfusion. Neurobiol Learn Mem 2013;101:46–54. 10.1016/j.nlm.2013.01.002 23313614

[28] Verma S, Li SH, Badiwala MV, Weisel RD, Fedak PW, Li RK, et al Endothelin antagonism and interleukin-6 inhibition attenuate the proatherogenic effects of C-reactive protein. Circulation 2002;105:1890–6. 11997273 10.1161/01.cir.0000015126.83143.b4

[29] Gombos T, Förhécz Z, Pozsonyi Z, Wallentin S, Papassotiriou J, Kunde J, et al Adrenomedullin and endothelin-1 are related to inflammation in chronic heart failure. Inflamm Res 2009;58:298–305. 10.1007/s00011-008-8184-5 19212702

